# On-Line Thickness Measurement for Two-Layer Systems on Polymer Electronic Devices

**DOI:** 10.3390/s131115747

**Published:** 2013-11-18

**Authors:** Ana Perez Grassi, Anton J. Tremmel, Alexander W. Koch, Hala J. El-Khozondar

**Affiliations:** 1 Institute for Measurement Systems and Sensor Technology, Technische Universität München, Theresienstr. 90/N5, Munich 80333, Germany; E-Mails: a.tremmel@tum.de (A.J.T.); a.w.koch@tum.de (A.W.K.); 2 Electrical Engineering Department, Islamic University of Gaza, Gaza P.O.Box 108, Palestine; E-Mail: hkhozondar@iugaza.edu

**Keywords:** optical films, TFI, thin film interferometry, two-layer model, TFR, thin film reflectance measurement, polymer electronics

## Abstract

During the manufacturing of printed electronic circuits, different layers of coatings are applied successively on a substrate. The correct thickness of such layers is essential for guaranteeing the electronic behavior of the final product and must therefore be controlled thoroughly. This paper presents a model for measuring two-layer systems through thin film reflectometry (TFR). The model considers irregular interfaces and distortions introduced by the setup and the vertical vibration movements caused by the production process. The results show that the introduction of these latter variables is indispensable to obtain correct thickness values. The proposed approach is applied to a typical configuration of polymer electronics on transparent and non-transparent substrates. We compare our results to those obtained using a profilometer. The high degree of agreement between both measurements validates the model and suggests that the proposed measurement method can be used in industrial applications requiring fast and non-contact inspection of two-layer systems. Moreover, this approach can be used for other kinds of materials with known optical parameters.

## 1. Introduction

The use of electronic functional polymers in the production of integrated circuits has been increasing significantly in recent years. Polymer electronics require new production techniques different from those used for silicon. The production of polymer electronics is based on a printing process similar to that used on paper. In particular, the circuit layers are successively printed on a substrate, which moves on a conveyor belt at a high velocity. The correct thickness of such layers is essential for guaranteeing the electrical behavior of the final product. Therefore, the thickness and other parameters must be monitored carefully during the production through a fast and non-contact process.

The conveyor belt of the printing setup complicates the implementation of transmission-based methods [1,2] for monitoring the film thickness. For this reason, we focused on reflection-based approaches. Common methods for measuring thin film thickness based on reflection include thin film interferometry (TFI) [3,4], thin film reflectometry (TFR) [5] and spectral ellipsometry [6]. Spectral ellipsometers can achieve a higher accuracy in thickness measurements than thin film reflectometers [7]. However, they require a more complex setup and are potentially slower [7]. TFI is based on a moving repetitive scanning process, which makes it only appropriate for static measurements [8,9]. As a result, TFR is advantageous for applications, such as on-line thickness monitoring, where measuring time should be kept short and/or the high accuracy of spectral ellipsometers is not needed.

The reflected signal measured by TFR is a function of the involved film thickness [5]. Therefore, by fitting it with a valid model, the thickness values can be obtained. A reflectance model for a single-layer system of polycrystalline silicon was presented by Hauge [10]. Hauge considers ideal interfaces for his model. However, in practice, irregular interfaces affect the reflectance significantly. The interfaces can be evaluated through the effective media approximation (EMA) [11] or by altering the Fresnel coefficients through different interface models [12]. Additionally, Montecchi [13] presents a model based on the perturbative method to measure thickness by considering inhomogeneities, roughness and slanted interfaces. This model is limited to one layer and discards perturbations on the layer-substrate and on the air-layer interfaces. Swanepoel [14] presents an approach to consider irregular interfaces on transmission spectra.

All mentioned contributions present models for single-layer systems. The most popular approaches for resolving multilayer systems are based on matrix methods [15] and recursive algorithms [16]. Most contributions in the inspection of multilayer systems are made for transmittance measurements [17,18] and X-ray reflectometry [16]. On the contrary, for white reflectometry, the literature lacks concrete works. From the reflectance expression of a single-layer system, like that given in [10], a two-layer reflectance model can be derived directly. Although the expression for a two-layer system is well known, the literature lacks models that can be directly applied to real measurements in white light reflectometry. Interface inhomogeneities and distortions introduced by the measurement equipment significantly affect the signal captured by the sensor. If the latter is neglected, the fitting process will compensate for these distortions with the thickness parameters. As a result, the model will match the measured signal for incorrect values.

Our approach uses Stearns' method [12] to incorporate the interface irregularities to the model. Stearns proposes to model the interface profile by using an analytical function. This method is largely used and well known in X-ray reflectometry [19,20]. However, to the best of our knowledge, its application in white light reflectometry for multilayer systems has not been published until now.

In the same way, we analyze the influence of the chromatic effect [21,22] introduced by the setup on the captured signal. This effect should be considered to avoid distortions in the measured results. Moreover, this model can be applied to measure other materials with known optical parameters.

## 2. Fundamentals of Thin Film Reflection

The complex refractive index, **n**(λ), of a material can be denoted as: **n**(λ) = *n*(λ) — *jk*(λ), where *n*(λ) is the index of refraction, *k*(λ) is the absorption coefficient and λ is the wavelength of the light. For the sake of simplicity, the dependency on the wavelength, λ, will be suppressed in the notation throughout this paper. In the case of normal incidence of light, the Fresnel coefficient, **r***_lm_*, for two successive films, *m* = *l* + 1, with refractive indices, **n***_l_* and **n***_m_*, respectively, is defined as follows [23]:

(1) $${\text{r}}_{lm}=\frac{{\text{n}}_{l}-{\text{n}}_{m}}{{\text{n}}_{l}+{\text{n}}_{m}}$$

The total reflection coefficient, **r**, of a single-layer system composed of a thin film (**n**_1_) on an absorbing substrate (**n**_2_) surrounded by air (**n**_0_) is given by [10]:

(2) $$\text{r}=\frac{{\text{r}}_{01}+{\text{r}}_{12}e^{-j{\text{d}}_{1}}}{1+{\text{r}}_{01}{\text{r}}_{12}e^{-j{\text{d}}_{1}}}$$

where **d**_1_ = 4π**n**_1_*d*_1_/λ is representing the thickness of the film. As given in Equation (1), **r**_01_ is the Fresnel coefficient between air and film and **r**_12_ between film and substrate. Finally, the reflectance, *R*(λ), is given by:

(3) $$R(\lambda )=\text{r}\;\cdot \;{\text{r}}^{*}$$

where r* indicates the complex conjugate of **r**.

**Figure 1.**
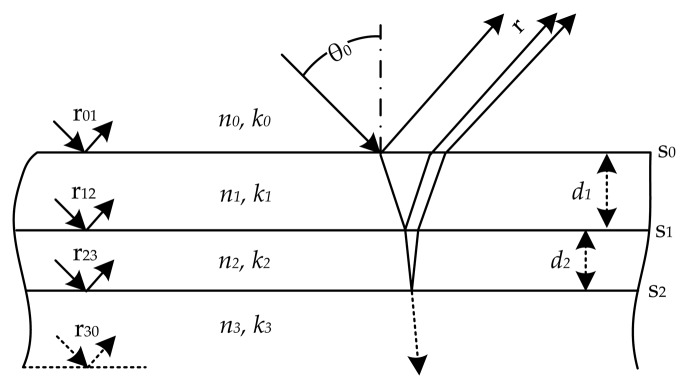
Two-layer system surrounded by air (*n*_0_, *k*_0_).

Figure 1 illustrates a two-layer system. In this case, two films (**n**_1_ and **n**_2_) are deposited successively on an absorbing substrate (**n**_3_). The whole system is surrounded by air (**n**_0_). To calculate the reflection coefficient for this system, Equation (2) can be extended as follows [24]:

(4) $$\text{r}=\frac{{\text{r}}_{01}+{\text{r}}_{12}e^{-j{\text{d}}_{1}}+{\text{r}}_{23}e^{-j\text{d}}+{\text{r}}_{01}{\text{r}}_{12}{\text{r}}_{23}e^{-j{\text{d}}_{2}}}{1+{\text{r}}_{01}{\text{r}}_{12}e^{-j{\text{d}}_{1}}+{\text{r}}_{12}{\text{r}}_{23}e^{-j{\text{d}}_{2}}+{\text{r}}_{01}{\text{r}}_{23}e^{-j\text{d}}}$$

where **d***_l_* = 4*π***n***_l_d_l_*/λ and **d** = **d**_1_ + **d**_2_. Again, by using Equation (3), the reflectance, *R*(λ), for a two-layer system with an absorbing substrate can be obtained.

## 3. Model Extension

Equation (4) describes a system with ideal interfaces between layers. However, in practice, irregular interfaces affect the reflectance and must be considered in the model. One approach to model interfaces is based on the effective media approximation (EMA) [11]. By EMA, the inhomogeneous interfaces between layers are replaced by fictitious homogeneous layers, which are incorporated as such in the model [25]. Another approach proposes to modify the Fresnel coefficients in order to reproduce the effect of the interfaces on the reflectance [12]. In this case, the Fresnel coefficients, **r***_lm_*, are altered by multiplying them with a function, *f*(*g_l_*), where *g_l_* assigns a thickness to the interface, *s_l_*, proportional to its grade of inhomogeneity. The modified Fresnel coefficients are defined as follows:

(5) $${\overset{ˇ}{\text{r}}}_{lm}={\text{r}}_{lm}\cdot f(g_{l})$$

Introducing Equation (5) in Equation (4), the modified reflection coefficient, **ř**, is obtained. This approach yields a simpler and faster solution than EMA, which makes it advantageous for our application. The form of *f*(*g_l_*) depends on the considered interface model. As explained in [12], *f*(*g_l_*) could be ideally defined if the exact three dimensional structure of the interface was known. In general, however, such detailed knowledge of the interface is unavailable, and it is more reasonable to model the interface profile using an analytical function. Four different interface functions are presented in [12].

The principal causes of interface inhomogeneities are: the roughness of a layer surface and the mix of materials originated when two layers came in contact. In the case of polymer electronics, the substrate surface is smooth and does not mix with the first applied layer. Therefore, we can consider the interface, **s**_2_ (Figure 1), as ideal. On the contrary, we cannot discard the presence of roughness and material mix on the interface, **s**_1_, between the first and second layer. In this case, *f*(*g*_1_) must model both kinds of inhomogeneities [16]. Finally, the reflection coefficient of the interface, **s**_0_, between air and the first layer is modified only by the surface roughness. In this case, *g*_0_ approximates the **RMS**value of the surface roughness [16]. For our measurements, we use roughness function *f*(*g_l_*) = exp (−2(2*πg_l_*/λ)^2^), which is a modification factor for the Fresnel reflection coefficient. It was generated by modeling the interface profile by an error function [12,16]. As suggested by Stearns, this function describes an interface produced by the diffusion of two materials. In most of the cases, *g_l_* gives only a qualitative description of the interface, but, as will be shown, its consideration is essential to measure the layer thickness correctly.

Equations (2) and (4) assume a completely absorbing or infinitely thick substrate. For thin transparent substrates, these assumptions are not valid. In this case, it might be necessary to consider the backside reflection coefficient, **r**_30_, at the interface between substrate and air (see Figure 1). This latter requires a recalculation of **r**. However, if the coherence length of light is much smaller than the axial dimensions of the substrate, the reflectance can be approximated as *R*(λ) = (**ř** · ř*) + *h*(*k*_3_, *d*_3_), where *h*(*k*_3_, *d*_3_) is defined as in [26]:

(6) $$h(k_{3},d_{3})=\{\begin{array}{ll} r_{30}^{2}, & k_{3}\to 0\\ 0, & k_{3}\gg 0\;\text{and}/\text{or}\;d_{3}\to \infty \end{array}$$

**Figure 2.**
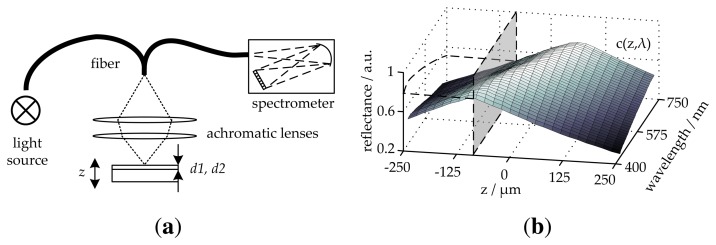
(**a**) Schematic representation of the measurement setup. The source light is coupled into a Y-branch fiber and guided to the measured object through the lenses, which introduced a chromatic effect. The multimode fiber has a numerical aperture of 0.22 and a diameter of 100 μm. Both lenses have a diameter of 12.5 mm and a focal length of 14 mm. The fill factor is 0.32. (**b**) Chromatic map, *c*(*z*, λ), for −250 ≤ *z* ≤ 250 *μ*m. The dashed lines show *c*(100 μm, λ).

Figure 2a illustrates our measurement setup. A complete description of this measurement system is presented in [21,22]. In this setup, light from a spectrally broad source is coupled to a multimode fiber (Y-branch) and guided by two focusing lenses to the layer system at normal incidence. The reflected light intensity is then coupled back into the fiber and led to a spectrometer. The focusing lenses introduce a focal shift in wavelength known as chromatic aberration [23], which affects the signal captured by the spectrometer. This aberration depends on the axial position, *z*, and wavelength λ. This distortion is important for our application, because the moving sample is affected by vertical vibration movements. These movements alter the variable, *z*, during the measurement and, therefore, also the form of the captured signal. To avoid the compensation of this effect by invalid thickness values during the fitting process, chromatic aberration must be considered in the model. The chromatic effect can be described through a function, *c*(*z*,λ), of the wavelength, λ, and the axial position, *z*, considering all system apertures. Figure 2b shows a set of chromatic functions for different values of *z* [22]. The chromatic effect on the reflectance can be described by multiplying both signals *R*(λ) · *c*(*z*,λ). The function, *c*(*z*, λ), can be obtained by modeling the setup or by performing a set of off-line measurements. In this work, *c*(*z*, λ) results from a set of measurements as described in [22]. By regenerating or recomputing *c*(*z*, λ), the model can be adapted for a new measurement setup or for new measurement conditions.

Now, we combine all described extensions to obtain a reflectance model for two-layer systems. Equation (4) gives an analytical expression for the total reflection, **r**, on absorbing substrates. If the Fresnel coefficients, **r***_lm_*, in Equation (4) are replaced by those **ř***_lm_* defined in Equation (5), we obtain an approximation of the total reflection denoted by **ř** that considers the selected interface models. From **ř**, a reflectance signal can be calculated using Equation (3). To consider the possibility of transparent substrates, function *h*(*k*_3_, *d*_3_), according to Equation (6), must be added. Finally, the complete result should be multiplied by *c*(*z*, λ) to include the chromatic effect described before:

(7) $$R(\lambda )=\left[\overset{ˇ}{\text{r}}\cdot {\overset{ˇ}{\text{r}}}^{*}+h(k_{3},d_{3})\right]\cdot c(z,\lambda )$$

Note that *R*(λ) denotes the modeled reflectance spectra, while *R*_0_(λ) describes the measured reflectance. Both spectra will be compared in the next section in order to find the thickness of the inspected layers.

## 4. Measurements and Results

### 4.1. Measured Samples

The analyzed samples are used in polymer electronic applications. They consist of PMMAand P3HTlayers applied successively by spinning on both transparent (PET) and non-transparent (Si) substrates. The optical parameters of all materials, *n*(λ) and *k*(λ), are well-known. Each sample presents a border, where each layer thickness can be measured by a profilometer. The device used is a Veeco, Dektak 6M Profilometer. The profilometer measurements provide the thickness reference values, $d_{1}^{0}$ and $d_{2}^{0}$, used to validate the proposed model. The layer application method generates a thickness variability of around ±10%. This should be considered, as the profilometer measurements were performed at the samples' edges, whereas the TFR measurements are performed in their centers. Table 1 lists all analyzed samples and their reference values. The first three samples have a non-transparent substrate of Si, and the last three have a transparent substrate of PET.

### 4.2. Algorithm, Conditions and Computational Times

The measured reflectance, *R*_0_(λ), is compared with the modeled one, *R*(λ), for different values of film thickness (*d*_1_, *d*_2_) interfaces (*g*_0_, *g*_1_)and axial displacement (*z*). By fitting both signals, the best parameters are selected in order to characterize the two inspected layers.

The starting points of the parameters and the searching intervals for the fitting algorithm must be correctly defined to achieve a valid result. This requires *a priori* knowledge of the minimum and maximum possible values for each parameter. This knowledge is based on, amongst others, the samples' materials, layer application method, measurement setup and experience.

The algorithm can be divided in two steps: the search for the initial points and the measurement fitting. The search for the initial points is performed with a recursive least squares algorithm. This is computationally intensive, but it allows larger searching intervals than other more efficient algorithms without falling in invalid minima. In this case, the limits of the searching intervals for the thickness values must not exceed ±50% of their real values. This means that the real values should be known *a priori* with a precision of ±50%, which is true for almost all practical industrial cases. The initial points are then precise enough to run the measurement fitting algorithm using smaller searching intervals, allowing us to use a Levenberg–Marquardt algorithm to solve the system.

The signal capture can be considered instant and not affected by the movement of the sample. The average fitting time is about 8.9 s, which allows us to control the production every 10 m. In the case of detecting failures on the thickness values, this rate gives a loss of material between 10 and 20 m, which is economically acceptable for these kinds of material. Moreover, if the production line guaranties no abrupt variations of the layers' thickness, the searching of the initial points can be performed off-line as part of a calibration process. This latter one reduces the measurement time considerably, allowing for a higher control frequency.

### 4.3. Results

**Figure 3.**
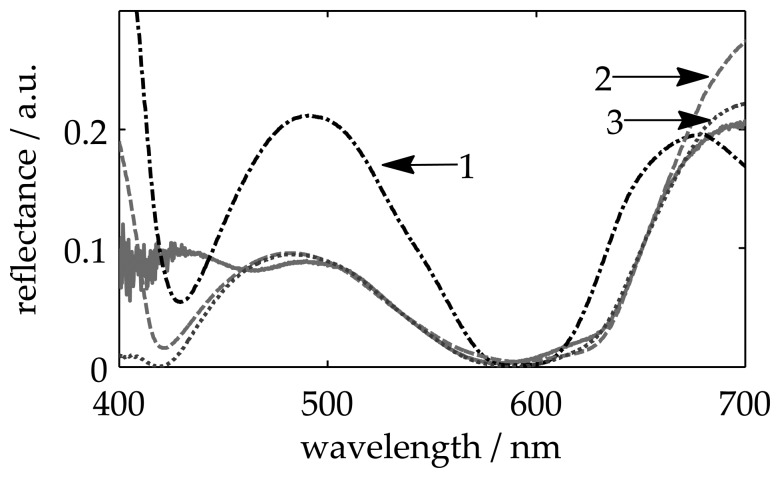
Comparison of different definitions of *R*(λ) for a two-layer system on the absorbing substrate. The solid line represents the measured reflectance, *R*_0_(λ). (**1**) *R*(λ) without considering inhomogeneity or chromatic effect; (**2**) *R*(λ) considering interface irregularities, but no chromatic function; (**3**) *R*(λ) considering roughness and chromatic function.

**Figure 4.**
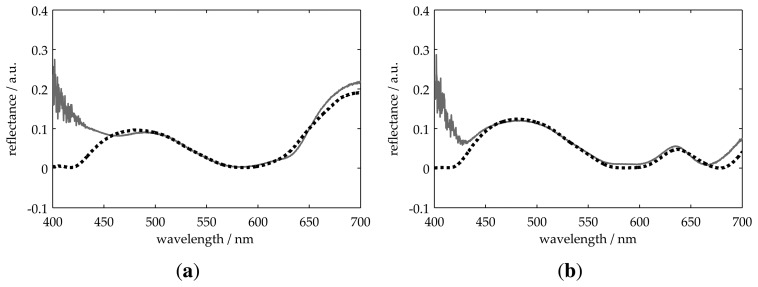
Measurement of two-layer systems on an absorbing substrate. The solid line represents the measured reflectance, and the dotted one is the best fitting result.

We use the first sample in Table 1 to show the influence of each model parameter on the measured values. The measured reflectance, *R*_0_(λ), for this sample is shown in Figure 3 through a solid line. In the first approach, we limit the model to *R*(λ) = **r** · r*, with r defined as in Equation (4), *i.e.*, without considering interface irregularities or chromatic effect. The resulting best fitting (curve 1 in Figure 3) gives thickness values for the first and second layer with deviations of 9.5% and 5% with respect to the reference values. In the second approach, we incorporate the interface function into the model. In this case, the Fresnel coefficients are modified as indicated in Equation (5), where *f*(*g_l_*)as defined as a roughness function [12,16] (the chromatic effect was not considered). Now, the best fitting (curve 2 in Figure 3) gives deviations of 1.6% and 8% for the first and second layer, respectively. Finally, we test the complete model described in Equation (7). The best fitting (curve 3 in Figure 3 and also in Figure 4a), which is shown in Table 1, gives deviations of less than 1.6% and 4% compared to the reference values.

Figures 4a through 6b show the measured *R*_0_(λ) and best fitting *R*(λ) reflectance for all samples presented in Table 1. For all measurements, the obtained thickness values present a deviation of less than 9% with respect to those obtained using the profilometer. These results validate our model for two-layer systems on both transparent and non-transparent substrates.

Additionally, although the parameters, *g*_0_, *g*_1_ and *z*, are considered in the model to approximate the values of *d*_1_ and *d*_2_ to the real ones, they also provide some extra information. Tactile measurements of the roughness in the first and second layer give mean peak to peak values of around 6.9 nm and 1.3 nm, respectively. The irregularities of the first interface, **s**_0_ (air-first layer), can be completely described by the roughness of the first layer. Therefore, as expected, the tactile measurement and the values of *g*_0_ are in the same order of magnitude. On the contrary, the tactile measurement for the second layer, which can only be performed by isolating it with respect to the first one, does not represent the interface, **s**_1_. For this interface, *g*_1_ describes a combination between roughness and interface inhomogeneity, which results in a significantly higher value than that given by the profilometer. Finally, the obtained values of *z* are inside the expected ones, and together with the obtained thickness, they can be used together to describe the setup configuration during the measurement.

## 5. Conclusion and Outlook

A model was proposed to measure the thickness of two-layer systems. This model was tested on samples used on polymer electronic applications; however, it can be easily extended to other kinds of surfaces with known optical characteristics. The model follows from an analytical derivation of the reflectance signal and is extended to consider surface interfaces, measuring setup characteristics and vertical vibration movements for absorbing and transparent substrates. Our results show that considering interface irregularities and chromatic effects increases the accuracy of the thickness measurements for both layers. Our method requires one to know a priori the expected value of the inspected thickness with a precision of ±50%, which is given for almost all real applications. The validity of the presented measurements was shown by comparing the obtained results with those of a profilometer device. The deviation between these two methods remains under the expected layer thickness deviation of 10%. The first interface parameter, *g*_0_, shows a meaningful match with the reference measurements. In the case of the second interface, the obtained value describes not only the roughness, but also the inhomogeneity, of the interface between both layers and cannot be compared with the profilometer results. The accuracy of this value will be checked in a future work by using an effective media approach. The presented results suggest that the proposed approach can be successfully used to measure the thicknesses of two-layer systems. The proposed method is especially adequate for applications in which a fast and non-contact inspection is required. This is the case, e.g., for the quality control of electronic components. An extension of this model to N-layer systems is also planned as future work.

## Figures and Tables

**Figure 5.**
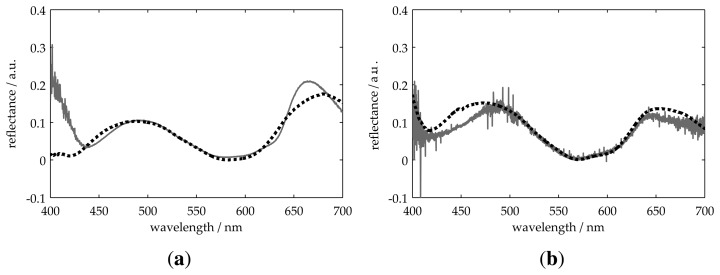
Measurement of two-layer systems on absorbing (**a**) and transparent (**b**) substrates. The solid line represents the measured reflectance, and the dotted one is the best fitting result.

**Figure 6.**
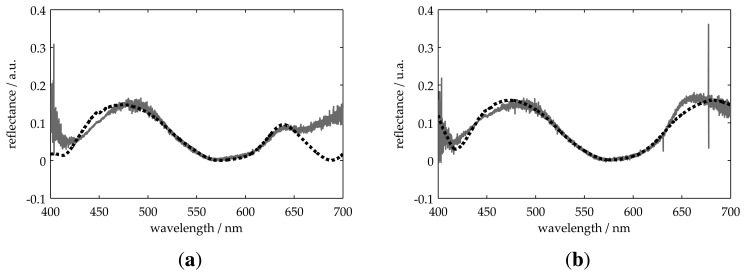
Measurement of two-layer systems on a transparent substrate. The solid line represents the measured reflectance, and the dotted one is the best fitting result.

**Table 1.** Best fitting results and reference values for two-layer systems on non-transparent (measurements 1–3) and transparent substrates (measurements 4–6). Values are in nanometers.

**Meas.**	*d*_1_	*d*_2_	*g*_0_	*g*_1_	$d_{1}^{0}$	$d_{2}^{0}$	*z*
1 (Figure 4a)	254	104	3	27	250	100	10 · 10^3^
2 (Figure 4b)	257	183	1	20	250	180	6 · 10^3^
3 (Figure 5a)	261	218	2	35	250	200	−46 · 10^3^
4 (Figure 5b)	254	152	2	32	250	150	−38 · 10^3^
5 (Figure 6a)	252	247	1	27	250	250	−85 · 10^3^
6 (Figure 6b)	252	192	3	31	250	190	−200 · 10^3^
